# Restoration models of flood resilient bridges: Survey data

**DOI:** 10.1016/j.dib.2021.107088

**Published:** 2021-04-23

**Authors:** Stergios Aristoteles Mitoulis, Sotirios A. Argyroudis

**Affiliations:** a University of Surrey, Department of Civil and Environmental Engineering, UK; b Brunel University London, Department of Civil and Environmental Engineering, UK

**Keywords:** Bridges, Restoration, Recovery, Resilience, Elicitation survey, Risk management, SDGs 9, 11, 13

## Abstract

The purpose of this survey is to define the restoration tasks after hydraulic-induced damage and/or loss of functionality of bridges. This includes the duration and sequence of restoration tasks, idle times, cost, and traffic/functionality loss for specified damage levels of given bridge components. The potential use of this data is the generation of sets of restoration and reinstatements functions for quantifying the resilience of bridges exposed to hydraulic hazards, i.e. scour, debris accumulation and hydraulic forces (Mitoulis et al. 2021). The data are expected to inform boroughs, county councils, road and rail owners and stakeholders by providing valuable information for managing efficiently their assets prior to and after catastrophic events on the basis of resilience. The survey was based on a questionnaire answered by experts on bridge and infrastructure engineering.

## Specifications Table

**Table:** 

Subject	Engineering; Civil and Structural Engineering
Specific subject area	Survey for riverine bridges exposed to flood effects; reinstatement and restoration for different damage levels; cost ratio and idle time; functionality and capacity loss; quantification of resilience for transport infrastructure
Type of data	Table supplemented with a questionnaire provided as supplementary file
How data were acquired	Survey by questionnaire, emails, and interviews
Data format	Raw Filtered based on logical tests
Parameters for data collection	The elicitation survey included input from five experts and another two interviews based in Europe. The condition for the selection of experts was for them to have background knowledge in bridge and/or geotechnical engineering and restoration.
Description of data collection	The collected data included: idle time, traffic capacity of the bridge after damage, prioritisation of restoration tasks, cost ratio, comments. The data concerned different damage levels and bridge components.
Data source location	United Kingdom, Greece, France, Norway
Data accessibility	With the article
Related research article	Mitoulis SA, Argyroudis S, Loli M, Imam B (2021). Restoration models for quantifying flood resilience of bridges. Engineering Structures, 238, 112180, https://doi.org/10.1016/j.engstruct.2021.112180

## Value of the Data

• The dataset contributes to bridging the inherent information gaps in bridge restoration after flood events and to better understand the interdependency between capacity and functionality (traffic).
• This data provides valuable information for researchers working in the area of infrastructure flood resilience, bridge owners and operators, consultants and risk assessors including the insurance sector, as well as for the advancement of existing and new regulations for climate resilience, design and management.
• Information provided in this dataset can be used for validation and calibration of relevant models, and resilience quantifications of critical highway and railway bridges.

## 1 Data Description

In this data brief we provide the answers elicitated from the experts based on the questionnaire which is provided in the supplementary data files. The survey includes questions regarding the recovery of bridges experiencing different damage level based on the following: (i) duration of restoration for 23 tasks; (ii) idle time; (iii) traffic capacity of the bridge after damage; (iv) prioritisation of restoration tasks; (v) cost ratio; (vi) comments from experts. Further details are included in Mitoulis et al. [1]. Similar approaches have been deployed in Lamb et al. [2] for assessing vulnerability factors for flood critical bridges and Misra et al. [3] on recovery of bridges exposed to diverse hazards. The significance of this data is that they enable the quantification of resilience for bridges and transport networks [4], [5], [6] in conjunction with recent research on the vulnerability of flood critical bridges [7].

**Fig. 1:**
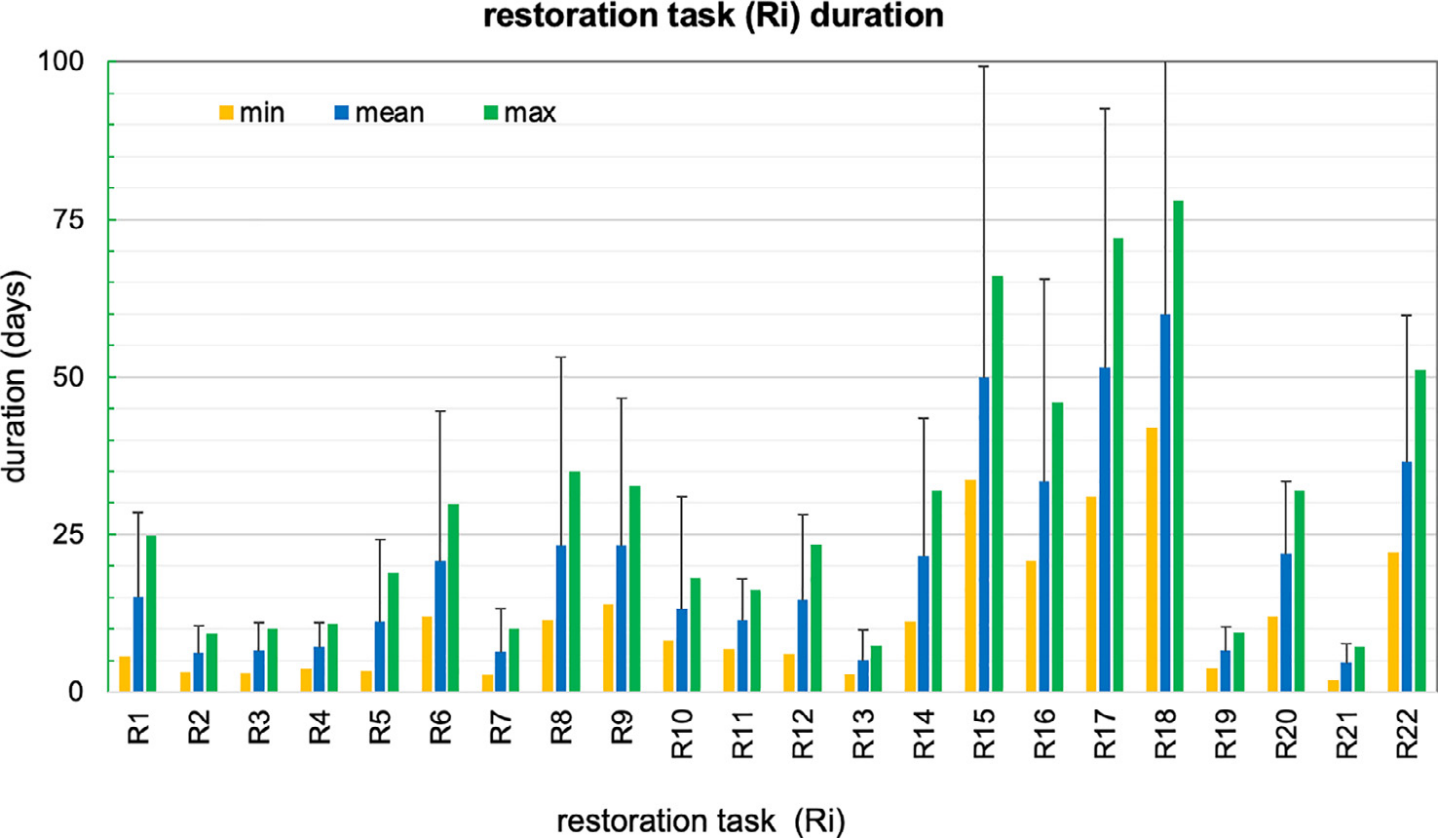
Duration of restoration tasks (minimum, mean and maximum values) and standard deviation of the mean value for 22 tasks based on experts’ answers. The duration for replacement of the bridge (R23) is given in the supplementary file.

All the answers of the experts are included in the supplementary files for spread and deep foundations experiencing minor, moderate, extensive and severe damage. Fig. 1 shows minimum, mean and maximum values of the durations of restoration tasks and standard deviation of the mean value. The same values are reported in the supplementary file, which also includes the answers for the fields (i) to (vi) described above.

## 2 Experimental Design, Materials and Methods

The experts were targeted considering their expertise and experience in bridge, geotechnical and infrastructure engineering and restoration works. The questionnaire is ongoing as it covers apart from foundations, other structural components of flood critical bridges, i.e. deck, bearings, piers, abutments and backfills. On two occasions, experts did not fill the questionnaire, however, they provided feedback verbally and this is reflected in Mitoulis et al. [1].

For future research, the data included in this article can be used to verify and extend recovery models for transport infrastructure, and in particular, for quantifying the climate resilience of riverine highway and railway bridges.

## Ethics Statement

The studies were performed in accordance with the relevant institutional and national regulations and legislation. Participants were requested to sign an informed consent form, after being informed of the aim of the survey.

Only primary data from datasets publicly available have been used for the development of the present dataset. All the relevant collaborations and funding sources have been mentioned.

## CRediT Author Statement

**Stergios Aristoteles Mitoulis:** Conceptualization, Methodology, Data curtion, Visualization, Writing original draft, Writing review & editing; **Sotirios A. Argyroudis:** Conceptualization, Methodology, Data curtion, Visualization, Writing original draft, Writing review & editing.

## Declaration of Competing Interest

The authors declare that they have no known competing financial interests or personal relationships which have or could be perceived to have influenced the work reported in this article.

## Appendix Supplementary materials

Image, application 1

Image, application 2
