# Supplementary material for: Restoration models of flood resilient bridges: Survey data
Source: Data Brief. 2021 Apr 23;36:107088. doi: 10.1016/j.dib.2021.107088 (PMC8114126; doi:10.1016/j.dib.2021.107088)
Supplement: Supplementary file 1 [file mmc1.docx]

Survey for bridge restoration after floods

Resilience of transport networks

**Stergios Aristoteles Mitoulis & Sotirios Argyroudis**

2021


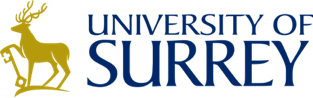


**Faculty of
Engineering & Physical Sciences**

**Department of Civil and**

**Environmental Engineering**

**Dr Stergios A Mitoulis**

E: s.mitoulis@surrey.ac.uk

Dear Expert,

**RE: Survey for bridge restoration after floods for generating resilience models**

The purpose of this survey is to define the restoration tasks after hydraulic-induced damages and/or loss of functionality of bridges in the framework of the ongoing research projects, which are available on <http://www.infrastructuresilience.com/projects/>, coordinated by the University of Surrey. The ultimate outcome of this survey will be the generation of a set of restoration functions for quantifying the resilience of bridges and transport networks in the UK, exposed to hydraulic hazards, i.e. scour, debris accumulation and hydraulic forces. This research is conducted for the first time in the international literature, and its findings are expected to inform boroughs, county councils, road and rail owners and stakeholders by providing valuable information for managing efficiently their assets prior and after catastrophic events on the basis of resilience.

To increase its robustness, this survey requires the participation of a pool of experts like yourself that will help us in reliably defining the restoration processes, accounting for the opinions of the larger technical community. To this end, the experts will provide their judgements by answering a series of questions asked in the questionnaire. We believe that your relevant expertise in hydraulic actions on transport infrastructure, and particular bridges, will provide valuable insights and thus contribute toward the generation of reliable restoration and resilience models.

We hereby formally invite you to be part of this research effort and we would be grateful if you could spare some time to fill-in the attached questionnaire. We estimate that your total time involvement for filling in the survey *will not exceed 30 min*. Your contribution will be officially acknowledged and highlighted in future scientific publications, unless otherwise stated. You will not be liable for any outcome. All the opinions provided by you will be treated in full confidence, and the results will be presented either in an aggregated way or in an anonymous format. The outcome of the expert judgment exercise process will be shared with you.

We very much appreciate your time and contribution to this important exercise by sharing your expertise. We also commit ourselves to acknowledge your contribution and share the results with you formally, if this is of interest to you and/or to your organisation.

If you have any other questions or comments on the survey, please email [s.mitoulis@surrey.ac.uk](mailto:s.mitoulis@surrey.ac.uk). The questionnaire is also available online: <http://www.infrastructuresilience.com/survey-for-bridge-restoration/>

Yours Sincerely,

**Dr Stergios Aristoteles Mitoulis**

**SURVEY FOR BRIDGE RESTORATION AFTER FLOODS**

Please provide your information in Table 1 before completing the survey:

**Table Expert’s details**

| **Name Surname** |  |
| --- | --- |
| **Organisation** |  |
| **Title/Position** |  |
| **e-mail** |  |
| **Address (optional)** |  |
| **Phone number (optional)** |  |
| **Rate your expertise on bridge restoration after floods from 0 to 10**  (0= no experience, 10=100+ hours of post-flood reconnaissance/repair) |  |
| **Professional experience** (years and areas of expertise) |  |
| **Consent to acknowledge your name in future publications and presentations** (yes/no) |  |
| **Consent to acknowledge your organisation name in future publications and presentations** (yes/no) |  |

1. **Instructions**

The survey for bridge restoration after floods is divided into **nine sections**:

1. Instructions,
2. Restoration tasks,
3. Quantification of the fragility and restoration of a 3-span pre-stressed concrete bridge,
4. Foundations,
5. Piers,
6. Abutments & wingwalls,
7. Bearings,
8. Deck,
9. Backfill & approach slab

You will be given the **"damage level"** (column 1 of Tables 3, 5, 7, 9, 11, 13, 15, 17, 19) of bridge components (for example a displacement or tilting of a pier causing 30-50 mm dislodgment of bearings and deck). Photographs or sketches illustrating the level of damage are provided in the supplementary tables of each section. Each damage level includes various damage modes, however, they may not occur all simultaneously, i.e., at least one damage mode can cause the corresponding damage level. After assessing this level of damage, please provide an **engineered estimation** of the following in the corresponding survey table of each section. These estimations can be based on your expert judgement, i.e. is not necessary to have real evidence from past events.

**Idle or lag time** (columns 2, 3 of Tables 3, 5, 7, 9, 11, 13, 15, 17, 19): Assuming that the restoration of the bridge is prioritised over others, please provide an estimate of the minimum and the maximum time before the initiation of any restoration work. This time might include, but is not limited to: emergency response, removal of standing water, inspection and condition assessment, site investigation, structural and foundation evaluation, design of measures, including organisational barriers. This time does not include any work or construction on the bridge.

**% traffic capacity (% of the normal bridge capacity)** (column 4 of Tables 3, 5, 7, 9, 11, 13, 15, 17, 19): The measure of the expected traffic capacity (0, 50 or 100 %) is the metric of "traffic restriction" for the bridge for each level of damage and for each point in time after the commencement of the restoration works (time: 0, 3, 15, 30, 60 days). For example, 3 days after the initiation of restoration works you may expect the bridge to be able to carry 50% of regular traffic capacity (i.e. has a traffic restriction of 50%). On day 0, the traffic capacity is linked solely on the structural capacity of the bridge structural components, except the case of the deck, which might include non-structural obstructions, e.g. accumulation of water or debris that obstruct the traffic. Thus, the traffic capacity on day 0 is the remaining capacity of the bridge before any restoration task commences. When you choose values for the % traffic capacity, consider only the effect of damage of that specific component to the functionality of the bridge, e.g. when considering bearings, assume that columns, footings, and abutments are intact.

Note that 0= full closure with the bridge having 0% traffic capacity, whilst 100= fully open corresponding to 100% traffic carrying capacity.

**Restoration task(s)** (column 5 of Tables 3, 5, 7, 9, 11, 13, 15, 17, 19): Choose and suggest the repair task that may be applied in order to recover the bridge component to its normal operation. Select the tasks **Ri** from the list given in Table 2, or identify a new one, and insert the corresponding repair task code to the survey table for each damage level. Please, provide your answers considering the reasonable order of the tasks, e.g. “R6, R5”, means that re-alignment of the pier (R6), is preceding the repair of cracks with epoxy (R5).

When completing the survey, assume that the typical post-disaster level of funding and resources are available for repair, and consider current best practices for repair procedures.

**Cost ratio** (column 6 of Tables 3, 5, 7, 9, 11, 13, 15, 17, 19): Provide an estimation for the cost of the repair tasks defined in column 5, as **a ratio of the construction cost of the entire bridge**. For example, repair cost ratio equal to 0.15 means that the cost of the restoration tasks is equal to 15% of the re-construction cost, or in other words, if the bridge cost is £3m, its restoration is £3m x 0.15= £450k.

**Comments:** Space is provided for comments in each survey table. Please comment on other component damage (deck, columns/piers, abutments, or foundation) that you might expect to see along with the component damage you are considering. Specify here if your estimations were based on a specific case-event. Any additional comments you may have throughout the process of completing the survey regarding the format, damage levels, bridge functionality, repair procedures, or other, will be very much appreciated.

1. **Restoration tasks for flood-induced bridge damages**

Please provide in the table below, an estimate of the minimum (column 3) and maximum (column 4) duration of the relevant restoration task shown in column 2. Task R0 doesn’t have duration as correspond to no action taken, Tasks R1 to R23, are pre-defined. If you identify additional tasks needed, please fill in the description of the restoration task and the corresponding duration.

**Table Restoration times for different restoration tasks**

| **code** | **restoration task** | **duration (days)** | |
| --- | --- | --- | --- |
|  |  | **minimum** | **maximum** |
| (1) | (2) | (3) | (4) |
| R0 | no action is required | *na* | *na* |
| R1 | armouring countermeasures and flow-altering/cofferdam |  |  |
| R2 | temporary support per pier |  |  |
| R3 | temporary support of one abutment |  |  |
| R4 | temporary support of one deck span /segment (midspan or support) |  |  |
| R5 | repair cracks and spalling with epoxy and/or concrete |  |  |
| R6 | re-alignment and/or leveling of pier |  |  |
| R7 | re-alignment of bearings |  |  |
| R8 | jacketing or local strengthening (pier or abutment or foundation) |  |  |
| R9 | jacketing or local strengthening (deck) |  |  |
| R10 | re-alignment of deck segment |  |  |
| R11 | erosion protection measures |  |  |
| R12 | rip-rap and/or gabions for filling of scour hole and scour protection |  |  |
| R13 | removal of debris |  |  |
| R14 | ground improvement per foundation |  |  |
| R15 | installation of deep foundation system |  |  |
| R16 | extension of foundation footing |  |  |
| R17 | reconstruction/replacement of the abutment and wingwalls |  |  |
| R18 | reconstruction/replacement of the pier |  |  |
| R19 | temporary support and replacement of the bearings |  |  |
| R20 | replacement of the backfill and approach slab and mudjacking |  |  |
| R21 | replacement of expansion joint |  |  |
| R22 | demolish/replacement of a deck span/segment |  |  |
| R23 | demolish/replacement (part) of the bridge |  |  |
| R24 | *please add customised task* |  |  |
| R25 | *please add customised task* |  |  |
| R26 | *please add customised task* |  |  |
| R27 | *please add customised task* |  |  |
| R28 | *please add customised task* |  |  |
| R29 | *please add customised task* |  |  |
| R30 | *please add customised task* |  |  |
|  |  |  |  |
|  |  |  |  |

1. **Quantification of the fragility and restoration of a 3-span pre-stressed concrete bridge**

This questionnaire envisages to cover the restoration tasks of any river crossing bridge with spread or piled foundations. The deck of the bridge is considered to be either continuous, e.g. box girder, or with a continuity slab, precast I-beams and continuity slab over the piers, i.e. no intermediate joints. The pier-to-deck & abutment-to-deck connections are considered to be either integral or through bearings. The number of spans and the geometry of each structural component were not considered in this questionnaire, due to the variability of these parameters. Indicatively, the reference bridge of this questionnaire has a total length of 101.5m and three equal spans of 33.5m. The deck has a total width of 13.5m. The height of the abutments is 8.0 m, the footing has a thickness of 1.0 m and is 5.5 m long. The piers have a height of 10.0 m. The shallow foundation footing has a thickness of 1.5 m and is 3.5 m long, as per the following figure. It is assumed that the bridge is of average importance, or specify in the comments if your estimations refer to different importance.

**Figure 1. The 3-span prestressed concrete bridge**

1. **Foundations**

Please provide your expert estimation for each level of damage in Tables 3 and 5 (see instructions in section 1).

**Table Restoration of hydraulic induced damage to spread foundations**

| **Damage level**  *(see Table 4 for description)* | **Idle time in days**  (before any restoration works) | | **Restoration time in days (after the initiation of the restoration works)** | | | | | | | | | | | | | | | **Restoration tasks & prioritisation** *(see Table 1, choose from the drop-down menu, add as many tasks as needed, considering a reasonable order)* | **Cost ratio**  *(% of replacement cost of the bridge)* |
| --- | --- | --- | --- | --- | --- | --- | --- | --- | --- | --- | --- | --- | --- | --- | --- | --- | --- | --- | --- |
|  |  |  | **0** | | | **3** | | | **15** | | | **30** | | | **60** | | |  |  |
|  | **min** | **max** | **% traffic capacity of the bridge after damage** | | | | | | | | | | | | | | |  |  |
| (1) | (2) | (3) | (4) (*check mark “X”*) | | | | | | | | | | | | | | | (5) | (6) |
|  |  |  | 0 | 50 | 100 | 0 | 50 | 100 | 0 | 50 | 100 | 0 | 50 | 100 | 0 | 50 | 100 |  |  |
| **Minor** |  |  |  |  |  |  |  |  |  |  |  |  |  |  |  |  |  | Choose an item. Choose an item.  Choose an item.  Choose an item.  Choose an item.  Choose an item.  Choose an item.  Choose an item. |  |
| **Moderate** |  |  |  |  |  |  |  |  |  |  |  |  |  |  |  |  |  | Choose an item.  Choose an item.  Choose an item.  Choose an item.  Choose an item.  Choose an item.  Choose an item.  Choose an item. |  |
| **Extensive** |  |  |  |  |  |  |  |  |  |  |  |  |  |  |  |  |  | Choose an item.  Choose an item.  Choose an item.  Choose an item.  Choose an item.  Choose an item.  Choose an item.  Choose an item.  Choose an item. |  |
| **Severe** |  |  |  |  |  |  |  |  |  |  |  |  |  |  |  |  |  | Choose an item.  Choose an item.  Choose an item.  Choose an item.  Choose an item.  Choose an item.  Choose an item.  Choose an item.  Choose an item.  Choose an item. |  |
| **Comments:** | | | | | | | | | | | | | | | | | | | |

**Table Description of damage levels for hydraulic induced damage to spread foundations**

| **Damage level** | **Description** | **Sketch** |
| --- | --- | --- |
| **Minor** | - Foundation settlement/sinking: < 20 mm - Foundation rotation/differential settlement: < 2‰ - Minor spalling (damage requires no more than cosmetic repair): crack width < 0.3mm - Scour hole depth and extent: 1.0D_f_ (where D_f_ is the foundation depth) - Safety Factor: > 3 | 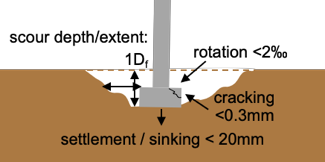 |
| **Moderate** | - Foundation settlement/sinking: 20-50 mm - Foundation rotation/differential settlement: 2-4‰ - Moderate cracking and spalling (foundation structurally still sound): crack width 0.3-0.6mm - Scour hole depth and extent: 1.0-1.5D_f_ - Safety Factor: 2-3 | 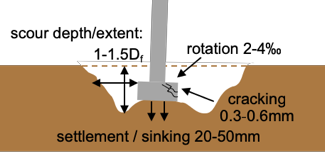 |
| **Extensive** | - Foundation settlement/sinking: 50-130 mm - Foundation rotation/differential settlement: 4-6‰ - Foundation degrading without collapse – shear failure (foundation structurally unsafe): crack width 0.6-3mm - Reinforcement yielding - Scour hole depth and extent: 1.5-2.0D_f_ - Safety Factor: 1-2 | 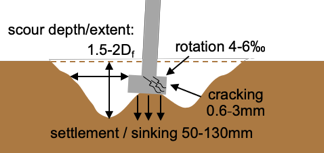 |
| **Complete** | - Foundation settlement/sinking: >130 mm - Foundation rotation/differential settlement: >6‰ - Overturning of the foundation: crack width >3mm - Reinforcement failure - Scour hole depth and extent: >2.0D_f_ - Safety Factor: <1 | 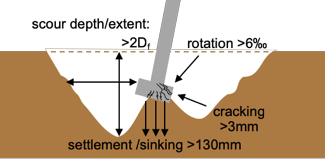 |

**Table Restoration of hydraulic induced damage to deep foundations**

| **Damage level**  *(see Table 6 for description)* | **Idle time in days**  (before any restoration works) | | **Restoration time in days (after the initiation of the restoration works)** | | | | | | | | | | | | | | | **Restoration tasks & prioritisation** *(see Table 1, choose from the drop-down menu, add as many tasks as needed, considering a reasonable order)* | **Cost ratio**  *(% of replacement cost of the bridge)* |
| --- | --- | --- | --- | --- | --- | --- | --- | --- | --- | --- | --- | --- | --- | --- | --- | --- | --- | --- | --- |
|  |  |  | **0** | | | **3** | | | **15** | | | **30** | | | **60** | | |  |  |
|  | **min** | **max** | **% traffic capacity of the bridge after damage** | | | | | | | | | | | | | | |  |  |
| (1) | (2) | (3) | (4) (*check mark “X”*) | | | | | | | | | | | | | | | (5) | (6) |
|  |  |  | 0 | 50 | 100 | 0 | 50 | 100 | 0 | 50 | 100 | 0 | 50 | 100 | 0 | 50 | 100 |  |  |
| **Minor** |  |  |  |  |  |  |  |  |  |  |  |  |  |  |  |  |  | Choose an item. Choose an item.  Choose an item.  Choose an item.  Choose an item.  Choose an item.  Choose an item.  Choose an item. |  |
| **Moderate** |  |  |  |  |  |  |  |  |  |  |  |  |  |  |  |  |  | Choose an item.  Choose an item.  Choose an item.  Choose an item.  Choose an item.  Choose an item.  Choose an item.  Choose an item. |  |
| **Extensive** |  |  |  |  |  |  |  |  |  |  |  |  |  |  |  |  |  | Choose an item.  Choose an item.  Choose an item.  Choose an item.  Choose an item.  Choose an item.  Choose an item.  Choose an item.  Choose an item. |  |
| **Severe** |  |  |  |  |  |  |  |  |  |  |  |  |  |  |  |  |  | Choose an item.  Choose an item.  Choose an item.  Choose an item.  Choose an item.  Choose an item.  Choose an item.  Choose an item.  Choose an item.  Choose an item. |  |
| **Comments:** | | | | | | | | | | | | | | | | | | | |

**Table Description of damage levels for hydraulic induced damage to deep foundations**

| **Damage level** | **Description** | **Sketch** |
| --- | --- | --- |
| **Minor** | - Buckling causing minor spalling and cracking: crack width < 0.3mm - Scour hole depth: 1.0D_pc_ (where D_pc_ is the foundation depth at the pile cap level) - Deep foundation settlement/sinking or pull-out: <20 mm - Deep foundation/pile cap rotation: <2‰ | *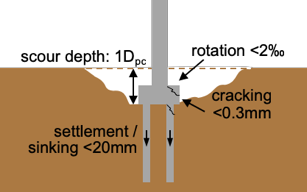* |
| **Moderate** | - Buckling causing moderate spalling and cracking: crack width 0.3-0.6mm - Soil is washed out, piles are revealed, scour hole depth: 1.0D_pc_ + 2d_p_ (where d_p_ is the pile diameter) - Deep foundation settlement/sinking or pull-out: 20-50mm - Deep foundation/pile cap rotation: 2-4‰ | 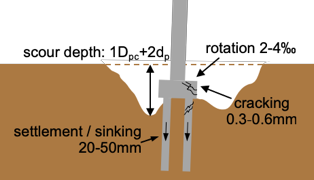 |
| **Extensive** | - Buckling causing extensive spalling and cracking: crack width 0.6-3mm - Soil is washed out, piles are revealed, scour hole depth: 1.0D_pc_ + 4d_p_ - Deep foundation settlement/sinking or pull-out: 50-130mm - Deep foundation/pile cap rotation: 4-6‰ - Reinforcement yielding | 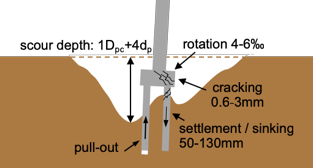 |
| **Complete** | - Buckling causing excessive cracking: crack width >3mm - Soil is washed out, piles are revealed, scour hole depth: 1.0D_pc_+ 6d_p_ - Deep foundation settlement/sinking or pull-out: >130mm - Deep foundation/pile cap rotation: >6‰ - Reinforcement failure | 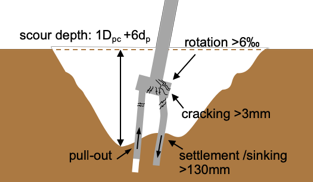 |

1. **Piers**

Please provide your expert estimation for each level of damage, in Table 7 (see instructions in section 1).

**Table Restoration of hydraulic induced damage to piers**

| **Damage level**  *(see Table 8 for description)* | **Idle time in days**  (before any restoration works) | | **Restoration time in days (after the initiation of the restoration works)** | | | | | | | | | | | | | | | **Restoration tasks & prioritisation** *(see Table 1, choose from the drop-down menu, add as many tasks as needed, considering a reasonable order)* | **Cost ratio**  *(% of replacement cost of the bridge)* |
| --- | --- | --- | --- | --- | --- | --- | --- | --- | --- | --- | --- | --- | --- | --- | --- | --- | --- | --- | --- |
|  |  |  | **0** | | | **3** | | | **15** | | | **30** | | | **60** | | |  |  |
|  | **min** | **max** | **% traffic capacity of the bridge after damage** | | | | | | | | | | | | | | |  |  |
| (1) | (2) | (3) | (4) (*check mark “X”*) | | | | | | | | | | | | | | | (5) | (6) |
|  |  |  | 0 | 50 | 100 | 0 | 50 | 100 | 0 | 50 | 100 | 0 | 50 | 100 | 0 | 50 | 100 |  |  |
| **Minor** |  |  |  |  |  |  |  |  |  |  |  |  |  |  |  |  |  | Choose an item. Choose an item.  Choose an item.  Choose an item.  Choose an item.  Choose an item.  Choose an item.  Choose an item. |  |
| **Moderate** |  |  |  |  |  |  |  |  |  |  |  |  |  |  |  |  |  | Choose an item.  Choose an item.  Choose an item.  Choose an item.  Choose an item.  Choose an item.  Choose an item.  Choose an item. |  |
| **Extensive** |  |  |  |  |  |  |  |  |  |  |  |  |  |  |  |  |  | Choose an item.  Choose an item.  Choose an item.  Choose an item.  Choose an item.  Choose an item.  Choose an item.  Choose an item.  Choose an item. |  |
| **Severe** |  |  |  |  |  |  |  |  |  |  |  |  |  |  |  |  |  | Choose an item.  Choose an item.  Choose an item.  Choose an item.  Choose an item.  Choose an item.  Choose an item.  Choose an item.  Choose an item.  Choose an item. |  |
| **Comments:** | | | | | | | | | | | | | | | | | | | |

**Table Description of damage levels for hydraulic induced damage to piers**

| **Damage level** | **Description** | **Sketch** |
| --- | --- | --- |
| **Minor** | - Pier settlement/sinking: <20 mm - Pier tilting: <2‰ - Minor spalling at the pier (damage requires no more than cosmetic repair), cracking width: <0.3mm | 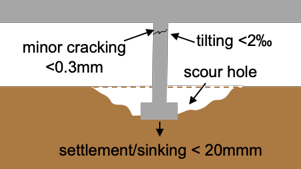 |
| **Moderate** | - Pier settlement/sinking: 20-50 mm - Pier tilting: 2-4‰ - Moderate shear cracking and spalling (pier structurally still sound), cracking width: 0.3-0.6mm | 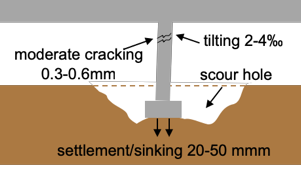 |
| **Extensive** | - Pier settlement/sinking: 50-130 mm - Pier tilting: 4-6‰ - Pier degrading without collapse – flexural and/or shear extensive damage (pier structurally unsafe), cracking width: 0.6-3mm - Reinforcement yielding | 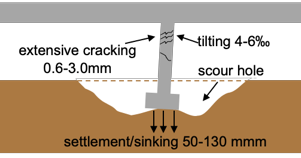 |
| **Complete** | - Pier settlement/sinking: >130 mm - Pier tilting: >6‰ - Flexural and/or shear failure and/or overturning of the pier, cracking width: >3mm - Reinforcement failure | 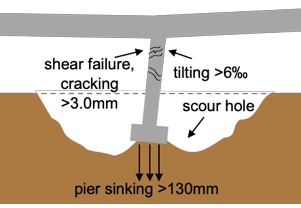 |

1. **Abutments & wingwalls**

Please provide your expert estimation for each level of damage in Table 9 (see instructions in section 1).

**Table Restoration of hydraulic induced damage to abutments and wingwalls**

| **Damage level**  *(see Table 10 for description)* | **Idle time in days**  (before any restoration works) | | **Restoration time in days (after the initiation of the restoration works)** | | | | | | | | | | | | | | | **Restoration tasks & prioritisation** *(see Table 1, choose from the drop-down menu, add as many tasks as needed, considering a reasonable order)* | **Cost ratio**  *(% of replacement cost of the bridge)* |
| --- | --- | --- | --- | --- | --- | --- | --- | --- | --- | --- | --- | --- | --- | --- | --- | --- | --- | --- | --- |
|  |  |  | **0** | | | **3** | | | **15** | | | **30** | | | **60** | | |  |  |
|  | **min** | **max** | **% traffic capacity of the bridge after damage** | | | | | | | | | | | | | | |  |  |
| (1) | (2) | (3) | (4) (*check mark “X”*) | | | | | | | | | | | | | | | (5) | (6) |
|  |  |  | 0 | 50 | 100 | 0 | 50 | 100 | 0 | 50 | 100 | 0 | 50 | 100 | 0 | 50 | 100 |  |  |
| **Minor** |  |  |  |  |  |  |  |  |  |  |  |  |  |  |  |  |  | Choose an item. Choose an item.  Choose an item.  Choose an item.  Choose an item.  Choose an item.  Choose an item.  Choose an item. |  |
| **Moderate** |  |  |  |  |  |  |  |  |  |  |  |  |  |  |  |  |  | Choose an item.  Choose an item.  Choose an item.  Choose an item.  Choose an item.  Choose an item.  Choose an item.  Choose an item. |  |
| **Extensive** |  |  |  |  |  |  |  |  |  |  |  |  |  |  |  |  |  | Choose an item.  Choose an item.  Choose an item.  Choose an item.  Choose an item.  Choose an item.  Choose an item.  Choose an item.  Choose an item. |  |
| **Severe** |  |  |  |  |  |  |  |  |  |  |  |  |  |  |  |  |  | Choose an item.  Choose an item.  Choose an item.  Choose an item.  Choose an item.  Choose an item.  Choose an item.  Choose an item.  Choose an item.  Choose an item. |  |
| **Comments:** | | | | | | | | | | | | | | | | | | | |

**Table Description of damage levels for hydraulic induced damage to abutments & wingwalls**

| **Damage level** | **Description** | **Sketch** |
| --- | --- | --- |
| **Minor** | - Abutment & wingwalls settlement/sinking: <20 mm - Abutment & wingwalls tilting: <2‰ - Minor spalling at the abutment & wingwalls (damage requires no more than cosmetic repair), cracking width: <0.3mm | 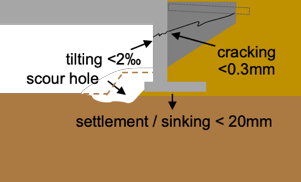 |
| **Moderate** | - Abutment & wingwalls settlement/sinking: 20-50 mm - Abutment & wingwalls tilting: 2-4‰ - Moderate shear cracking and spalling (abutment structurally still sound), cracking width: 0.3-0.6mm | 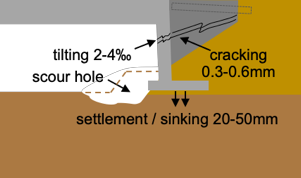 |
| **Extensive** | - Abutment & wingwalls settlement/sinking: 50-130 mm - Abutment & wingwalls tilting: 4-6‰ - Abutment & wingwalls degrading without collapse – flexural and/or shear extensive damage (abutment structurally unsafe), cracking width: 0.6-3mm - Reinforcement yielding | 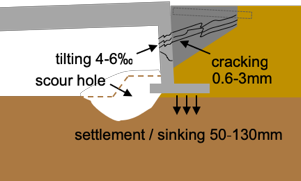 |
| **Complete** | - Abutment & wingwalls settlement/sinking: >130 mm - Abutment & wingwalls tilting: >6‰ - Flexural and/or shear failure and/or overturning of the abutment & wingwalls, cracking width: >3mm - Reinforcement failure | 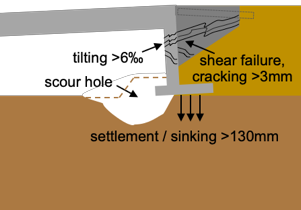 |

1. **Bearings**

Please provide your expert estimation for each level of damage in Table 11 (see instructions in section 1).

**Table Restoration of hydraulic induced damage to bearings**

| **Damage level**  *(see Table 12 for description)* | **Idle time in days**  (before any restoration works) | | **Restoration time in days (after the initiation of the restoration works)** | | | | | | | | | | | | | | | **Restoration tasks & prioritisation** *(see Table 1, choose from the drop-down menu, add as many tasks as needed, considering a reasonable order)* | **Cost ratio**  *(% of replacement cost of the bridge)* |
| --- | --- | --- | --- | --- | --- | --- | --- | --- | --- | --- | --- | --- | --- | --- | --- | --- | --- | --- | --- |
|  |  |  | **0** | | | **3** | | | **15** | | | **30** | | | **60** | | |  |  |
|  | **min** | **max** | **% traffic capacity of the bridge after damage** | | | | | | | | | | | | | | |  |  |
| (1) | (2) | (3) | (4) (*check mark “X”*) | | | | | | | | | | | | | | | (5) | (6) |
|  |  |  | 0 | 50 | 100 | 0 | 50 | 100 | 0 | 50 | 100 | 0 | 50 | 100 | 0 | 50 | 100 |  |  |
| **Minor** |  |  |  |  |  |  |  |  |  |  |  |  |  |  |  |  |  | Choose an item.  Choose an item.  Choose an item.  Choose an item.  Choose an item.  Choose an item.  Choose an item.  Choose an item. |  |
| **Moderate** |  |  |  |  |  |  |  |  |  |  |  |  |  |  |  |  |  | Choose an item.  Choose an item.  Choose an item.  Choose an item.  Choose an item.  Choose an item.  Choose an item.  Choose an item. |  |
| **Extensive** |  |  |  |  |  |  |  |  |  |  |  |  |  |  |  |  |  | Choose an item.  Choose an item.  Choose an item.  Choose an item.  Choose an item.  Choose an item.  Choose an item.  Choose an item.  Choose an item. |  |
| **Severe** |  |  |  |  |  |  |  |  |  |  |  |  |  |  |  |  |  | Choose an item.  Choose an item.  Choose an item.  Choose an item.  Choose an item.  Choose an item.  Choose an item.  Choose an item.  Choose an item.  Choose an item. |  |
| **Comments:** | | | | | | | | | | | | | | | | | | | |

**Table Description of damage levels for hydraulic induced damage to bearings**

| **Damage level** | **Description** | **Sketch** |
| --- | --- | --- |
|  |  | **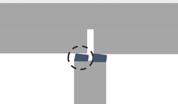** |
| **Minor** | - Bearing shear displacement: <20mm - Bearing rotation: <3‰ - Bearing axial displacement: <5mm - Tensile stress 0-0.5MPa |  |
| **Moderate** | - Bearing shear displacement: 20-50mm - Bearing rotation: 3-6‰ - Bearing axial displacement: 5-10mm - Tensile stress 0.5-1.0MPa |  |
| **Extensive** | - Bearing shear displacement: 50-100mm - Bearing rotation: 6-12‰ - Bearing axial displacement: 10-20mm - Tensile stress 1-1.5MPa | ** |
| **Complete** | - Bearing shear displacement: >100mm - Bearing rotation: >12‰ - Bearing axial displacement: >20mm - Tensile stress >1.5 MPa |  |

1. **Deck**

Please provide your expert estimation for each level of damage in Tables 13,15, and 17 (see instructions in section 1).

**Table Restoration of hydraulic induced damage to simply-supported deck**

| **Damage level**  *(see Table 14 for description)* | **Idle time in days**  (before any restoration works) | | **Restoration time in days (after the initiation of the restoration works)** | | | | | | | | | | | | | | | **Restoration tasks & prioritisation** *(see Table 1, choose from the drop-down menu, add as many tasks as needed, considering a reasonable order)* | **Cost ratio**  *(% of replacement cost of the bridge)* |
| --- | --- | --- | --- | --- | --- | --- | --- | --- | --- | --- | --- | --- | --- | --- | --- | --- | --- | --- | --- |
|  |  |  | **0** | | | **3** | | | **15** | | | **30** | | | **60** | | |  |  |
|  | **min** | **max** | **% traffic capacity of the bridge after damage** | | | | | | | | | | | | | | |  |  |
| (1) | (2) | (3) | (4) (*check mark “X”*) | | | | | | | | | | | | | | | (5) | (6) |
|  |  |  | 0 | 50 | 100 | 0 | 50 | 100 | 0 | 50 | 100 | 0 | 50 | 100 | 0 | 50 | 100 |  |  |
| **Minor** |  |  |  |  |  |  |  |  |  |  |  |  |  |  |  |  |  | Choose an item. Choose an item.  Choose an item.  Choose an item.  Choose an item.  Choose an item.  Choose an item.  Choose an item. |  |
| **Moderate** |  |  |  |  |  |  |  |  |  |  |  |  |  |  |  |  |  | Choose an item.  Choose an item.  Choose an item.  Choose an item.  Choose an item.  Choose an item.  Choose an item.  Choose an item. |  |
| **Extensive** |  |  |  |  |  |  |  |  |  |  |  |  |  |  |  |  |  | Choose an item.  Choose an item.  Choose an item.  Choose an item.  Choose an item.  Choose an item.  Choose an item.  Choose an item.  Choose an item. |  |
| **Severe** |  |  |  |  |  |  |  |  |  |  |  |  |  |  |  |  |  | Choose an item.  Choose an item.  Choose an item.  Choose an item.  Choose an item.  Choose an item.  Choose an item.  Choose an item.  Choose an item.  Choose an item. |  |
| **Comments:** | | | | | | | | | | | | | | | | | | | |

**Table Description of damage levels for hydraulic induced damage to simply-supported deck**

| **Damage level** | **Description** | **Sketch** |
| --- | --- | --- |
| **Minor** | - Minor spalling and cracking of the deck, cracking width: <0.3mm - Vertical and/or horizontal deflections/displacements of the deck: <40mm | 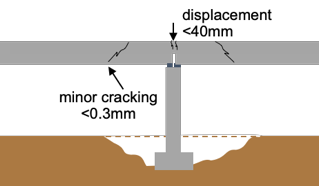 |
| **Moderate** | - Moderate spalling and cracking of the deck, cracking width: 0.3-0.6mm - Vertical and/or horizontal deflections/displacements of the deck: 40-80mm - Twisting/rotation of the deck about longitudinal axis: <2‰ | 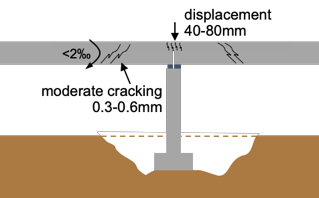 |
| **Extensive** | - Extensive spalling and cracking of the deck, cracking width: 0.6-3mm - Vertical and/or horizontal deflections/displacements of the deck: 80-200mm - Twisting/rotation of the deck about longitudinal axis: 2-8‰ - Reinforcement or prestressed steel yields in one location - Span (partial) unseating at one support | 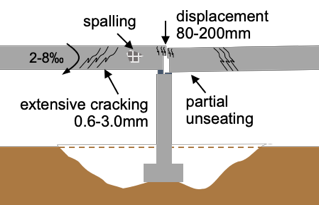 |
| **Complete** | - Excessive spalling and cracking of the deck, cracking width: >3mm - Vertical and/or horizontal deflections/displacements >2000mm - Twisting/rotation of the deck about longitudinal axis: >8‰ - Reinforcement or prestressed steel fails in multiple locations - Span unseating | 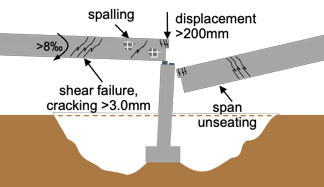 |

**Table Restoration of hydraulic induced damage to continuous deck**

| **Damage level**  *(see Table 16 for description)* | **Idle time in days**  (before any restoration works) | | **Restoration time in days (after the initiation of the restoration works)** | | | | | | | | | | | | | | | **Restoration tasks & prioritisation** *(see Table 1, choose from the drop-down menu, add as many tasks as needed, considering a reasonable order)* | **Cost ratio**  *(% of replacement cost of the bridge)* |
| --- | --- | --- | --- | --- | --- | --- | --- | --- | --- | --- | --- | --- | --- | --- | --- | --- | --- | --- | --- |
|  |  |  | **0** | | | **3** | | | **15** | | | **30** | | | **60** | | |  |  |
|  | **min** | **max** | **% traffic capacity of the bridge after damage** | | | | | | | | | | | | | | |  |  |
| (1) | (2) | (3) | (4) (*check mark “X”*) | | | | | | | | | | | | | | | (5) | (6) |
|  |  |  | 0 | 50 | 100 | 0 | 50 | 100 | 0 | 50 | 100 | 0 | 50 | 100 | 0 | 50 | 100 |  |  |
| **Minor** |  |  |  |  |  |  |  |  |  |  |  |  |  |  |  |  |  | Choose an item. Choose an item.  Choose an item.  Choose an item.  Choose an item.  Choose an item.  Choose an item.  Choose an item. |  |
| **Moderate** |  |  |  |  |  |  |  |  |  |  |  |  |  |  |  |  |  | Choose an item.  Choose an item.  Choose an item.  Choose an item.  Choose an item.  Choose an item.  Choose an item.  Choose an item. |  |
| **Extensive** |  |  |  |  |  |  |  |  |  |  |  |  |  |  |  |  |  | Choose an item.  Choose an item.  Choose an item.  Choose an item.  Choose an item.  Choose an item.  Choose an item.  Choose an item.  Choose an item. |  |
| **Severe** |  |  |  |  |  |  |  |  |  |  |  |  |  |  |  |  |  | Choose an item.  Choose an item.  Choose an item.  Choose an item.  Choose an item.  Choose an item.  Choose an item.  Choose an item.  Choose an item.  Choose an item. |  |
| **Comments:** | | | | | | | | | | | | | | | | | | | |

**Table Description of damage levels for hydraulic induced damage to continuous deck**

| **Damage level** | **Description** | **Sketch** |
| --- | --- | --- |
| **Minor** | - Minor spalling and cracking of the deck, cracking width: < 0.3mm - Vertical and/or horizontal deflections/displacements of the deck: <20mm | 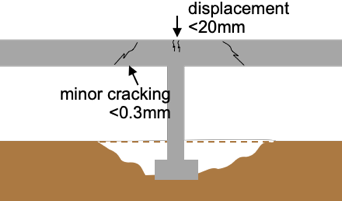 |
| **Moderate** | - Moderate spalling and cracking of the deck, cracking width: 0.3-0.6mm - Vertical and/or horizontal deflections/displacements of the deck: 20-50mm - Twisting/rotation of the deck about longitudinal axis: <1‰ | 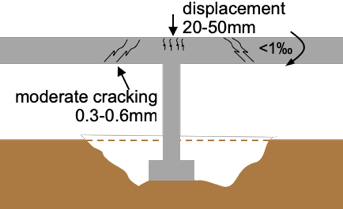 |
| **Extensive** | - Extensive spalling and cracking of the deck, cracking width: 0.6-3mm - Vertical and/or horizontal deflections/displacements of the deck: 50-130mm - Twisting/rotation of the deck about longitudinal axis: 1-4‰ - Reinforcement or prestressed steel yields in one location and/or hinge formation at one location | 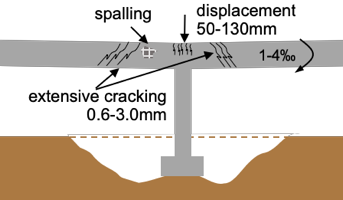 |
| **Complete** | - Excessive spalling and cracking of the deck, cracking width: >3mm - Vertical and/or horizontal deflections/displacements >130mm - Twisting/rotation of the deck about longitudinal axis: >4‰ - Reinforcement or prestressed steel yields in multiple locations and/or span collapses | 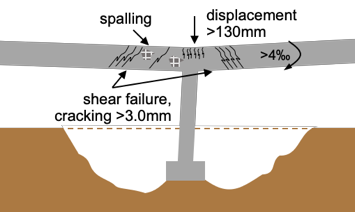 |

**Table Restoration of hydraulic induced disruptions to bridge deck**

| **Damage level**  *(see Table 18 for description)* | **Idle time in days**  (before any restoration works) | | **Restoration time in days (after the initiation of the restoration works)** | | | | | | | | | | | | | | | **Restoration tasks & prioritisation** *(see Table 1, choose from the drop-down menu, add as many tasks as needed, considering a reasonable order)* | **Cost ratio**  *(% of replacement cost of the bridge)* |
| --- | --- | --- | --- | --- | --- | --- | --- | --- | --- | --- | --- | --- | --- | --- | --- | --- | --- | --- | --- |
|  |  |  | **0** | | | **3** | | | **15** | | | **30** | | | **60** | | |  |  |
|  | **min** | **max** | **% traffic capacity of the bridge after damage** | | | | | | | | | | | | | | |  |  |
| (1) | (2) | (3) | (4) (*check mark “X”*) | | | | | | | | | | | | | | | (5) | (6) |
|  |  |  | 0 | 50 | 100 | 0 | 50 | 100 | 0 | 50 | 100 | 0 | 50 | 100 | 0 | 50 | 100 |  |  |
| **Minor** |  |  |  |  |  |  |  |  |  |  |  |  |  |  |  |  |  | Choose an item. Choose an item.  Choose an item.  Choose an item.  Choose an item.  Choose an item.  Choose an item.  Choose an item. |  |
| **Moderate** |  |  |  |  |  |  |  |  |  |  |  |  |  |  |  |  |  | Choose an item.  Choose an item.  Choose an item.  Choose an item.  Choose an item.  Choose an item.  Choose an item.  Choose an item. |  |
| **Extensive** |  |  |  |  |  |  |  |  |  |  |  |  |  |  |  |  |  | Choose an item.  Choose an item.  Choose an item.  Choose an item.  Choose an item.  Choose an item.  Choose an item.  Choose an item.  Choose an item. |  |
| **Severe** |  |  |  |  |  |  |  |  |  |  |  |  |  |  |  |  |  | Choose an item.  Choose an item.  Choose an item.  Choose an item.  Choose an item.  Choose an item.  Choose an item.  Choose an item.  Choose an item.  Choose an item. |  |
| **Comments:** | | | | | | | | | | | | | | | | | | | |

**Table Description of functionality loss levels for hydraulic induced disruptions to bridge deck**

| **Functionality loss level** | **Description** | **Sketch** |
| --- | --- | --- |
| **Minor** | - Accumulation of water due to overtopping, after extensive rainfall or flash flood: depth of water <50mm - Accumulation of debris due to landsliding of adjacent slopes or flooding: thickness of debris layer* <20mm |  |
| **Moderate** | - Accumulation of water due to overtopping, after extensive rainfall or flash flood: depth of water 50-125mm - Accumulation of debris due to landsliding of adjacent slopes or flooding: thickness of debris layer 20-50mm |  |
| **Extensive** | - Accumulation of water due to overtopping, after extensive rainfall or flash flood: depth of water 125-300mm - Accumulation of debris due to landsliding of adjacent slopes or flooding: thickness of debris layer 50-100mm - Extensive deterioration of the pavement - Extensive degradation of road markings and signage (poles, barriers, etc) |  |
| **Excessive** | - Accumulation of water due to overtopping, after extensive rainfall or flash flood: depth of water >300mm - Accumulation of debris due to landsliding of adjacent slopes or flooding: thickness of debris layer >100mm - Excessive deterioration of the pavement - Failure of road markings and signage (poles, barriers, etc) | 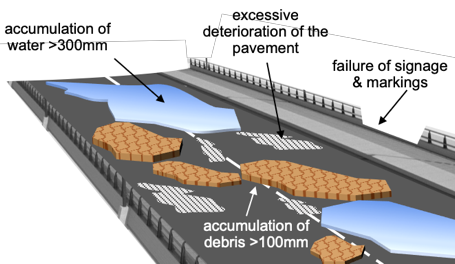 |
| * The thickness of debris corresponds to the equivalent average thickness of debris on the entire area of the deck if this was uniformly distributed | | |

1. **Backfill and approach slab**

Please provide your expert estimation for each level of damage in Table 19 (see instructions in section 1).

**Table Restoration of hydraulic induced damage to backfill and approach slab**

| **Damage level**  *(see Table 20 for description)* | **Idle time in days**  (before any restoration works) | | **Restoration time in days (after the initiation of the restoration works)** | | | | | | | | | | | | | | | **Restoration tasks & prioritisation** *(see Table 1, choose from the drop-down menu, add as many tasks as needed, considering a reasonable order)* | **Cost ratio**  *(% of replacement cost of the bridge)* |
| --- | --- | --- | --- | --- | --- | --- | --- | --- | --- | --- | --- | --- | --- | --- | --- | --- | --- | --- | --- |
|  |  |  | **0** | | | **3** | | | **15** | | | **30** | | | **60** | | |  |  |
|  | **min** | **max** | **% traffic capacity of the bridge after damage** | | | | | | | | | | | | | | |  |  |
| (1) | (2) | (3) | (4) (*check mark “X”*) | | | | | | | | | | | | | | | (5) | (6) |
|  |  |  | 0 | 50 | 100 | 0 | 50 | 100 | 0 | 50 | 100 | 0 | 50 | 100 | 0 | 50 | 100 |  |  |
| **Minor** |  |  |  |  |  |  |  |  |  |  |  |  |  |  |  |  |  | Choose an item. Choose an item.  Choose an item.  Choose an item.  Choose an item.  Choose an item.  Choose an item.  Choose an item. |  |
| **Moderate** |  |  |  |  |  |  |  |  |  |  |  |  |  |  |  |  |  | Choose an item.  Choose an item.  Choose an item.  Choose an item.  Choose an item.  Choose an item.  Choose an item.  Choose an item. |  |
| **Extensive** |  |  |  |  |  |  |  |  |  |  |  |  |  |  |  |  |  | Choose an item.  Choose an item.  Choose an item.  Choose an item.  Choose an item.  Choose an item.  Choose an item.  Choose an item.  Choose an item. |  |
| **Severe** |  |  |  |  |  |  |  |  |  |  |  |  |  |  |  |  |  | Choose an item.  Choose an item.  Choose an item.  Choose an item.  Choose an item.  Choose an item.  Choose an item.  Choose an item.  Choose an item.  Choose an item. |  |
| **Comments:** | | | | | | | | | | | | | | | | | | | |

**Table Damage levels for hydraulic induced damage to backfill and approach slab**

| **Damage level** | **Description** | **Sketch** |
| --- | --- | --- |
| **Minor** | - Backfill and approach slab settlement: <25mm - Minor cracking of the approach slab: <0.6mm | 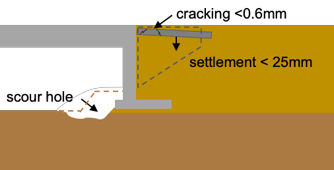 |
| **Moderate** | - Backfill and approach slab settlement: 25-150mm - Moderate cracking of the approach slab: 0.6-1.2mm - Moderate scour or wash out of the backfill: ~10% loss of the backfill material (i.e. volume) | 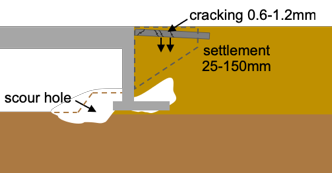 |
| **Extensive** | - Backfill and approach slab settlement: 150-400 mm - Extensive cracking of the approach slab: 1.2-6mm - Extensive scour or wash out of the backfill: ~25% loss of the backfill material (i.e. volume) | 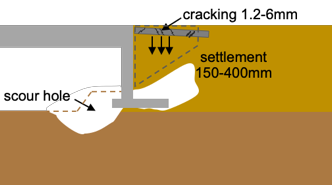 |
| **Complete** | - Backfill and approach slab settlement: > 400mm - Excessive cracking of the approach slab: >6mm - Excessive scour or wash out of the backfill: >25% loss of the backfill material (i.e. volume) | 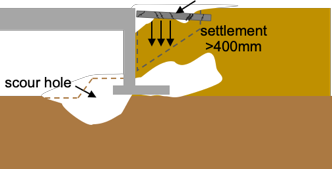 |
